# Factors Influencing Expatriate Adjustment in Qatar’s Healthcare Workforce: A Cross-Sectional Study

**DOI:** 10.7759/cureus.77044

**Published:** 2025-01-06

**Authors:** Ayesha Bashir

**Affiliations:** 1 International Human Resource Managment, University of Edinburgh, Edinburgh, GBR

**Keywords:** cultural integration, expatriate adjustment, multifactorial analysis, professional adaptation, qatar healthcare

## Abstract

Background: The healthcare industry in the Middle East significantly depends on expatriate professionals, whose ability to adjust to the local socio-cultural environment is critical for job satisfaction and, ultimately, the quality of patient care. This study aims to identify factors influencing expatriate adjustment within Qatar's healthcare sector.

Methodology: A comprehensive cross-sectional questionnaire survey was conducted among 385 healthcare workers in Qatar, utilizing expatriate-focused WhatsApp groups for dissemination. The data collection took place from October 1 to December 31, 2022. The survey assessed various elements influencing expatriate adjustment, measuring both adjustment and social support using a five-point Likert scale, ranging from “Extremely Adjusted” to “Extremely Disadjusted” for adjustment and from “No one would support” to “Several would support” for social support.

Results: The response rate was 51.69% (199/385), with participants representing 10 nationalities, predominantly aged 35-44 years and having a tenure of four to seven years in Qatar. Nurses and doctors comprised 83.9% of the respondents. Notably, females reported better adjustment (35.2% “Extremely Adjusted”) compared to males (28.7%). Age significantly impacted adjustment, with the 35-44 age group exhibiting the highest proportion of “Extremely Adjusted” individuals (36.4%). While trends were observed regarding nationality and length of stay, they were not statistically significant. Proficiency in Arabic was positively correlated with adjustment (p = 0.053), particularly among fluent speakers (27.4% “Extremely Adjusted”). Social support was found to be crucial (p = 0.001), with a lack of support associated with a high rate of “Extremely Disadjusted” outcomes (72.7%).

Conclusion: This study highlights the key factors affecting expatriate adjustment in Qatar's healthcare sector, emphasizing the significance of cultural assimilation and social networks. Further research is warranted to explore targeted interventions that can facilitate expatriates' integration into social and professional networks, thereby enhancing their capacity to deliver high-quality healthcare services.

## Introduction

Healthcare expatriate workers in the Middle East occupy a critical role, bringing indispensable expertise to the region’s evolving medical sector. Their diverse professional backgrounds and international experience are vital in addressing the expanding healthcare needs, contributing specialized knowledge, and advancing patient care [1]. However, their effectiveness hinges significantly on successful cultural and professional adjustment. Navigating a new set of cultural norms and healthcare practices requires adaptability and cultural sensitivity, essential for seamless integration into the local healthcare system. In addition to enriching the medical workforce with their clinical skills, expatriates also facilitate cross-cultural exchanges and the adoption of global best practices [2]. Their adjustment to the local environment is crucial for their personal and professional fulfillment and for enhancing the quality and scope of healthcare services, thus playing a pivotal role in the continuous advancement of healthcare standards in the Middle East. Effective adjustment is key for expatriates' personal and professional success, underscoring the need for well-structured orientation and integration programs by employers. In Qatar's healthcare sector, with its diverse cultural and demographic settings, such comprehensive support for expatriates is especially crucial for smooth integration [3].

Qatar's rapid development, fueled by significant economic growth and heightened global presence, notably through hosting the 2022 FIFA World Cup, has established it as a hub for expatriate professionals [4]. The World Cup event was not merely a sporting milestone but a transformative agent in Qatar's societal and economic landscape, notably influencing workforce diversification across various sectors, with the healthcare sector being a salient example. This period of dynamic change in Qatar's healthcare, incorporating both public and private health initiatives, has been particularly responsive to its growing population's evolving demographics and health needs. The influx of international visitors during the World Cup further accentuated the necessity for an adaptable and resilient healthcare infrastructure that can cater to both local and international health demands [4,5]. The integration of expatriate health professionals into Qatar's healthcare system has been essential in expanding its capacity and versatility, ensuring that it can effectively navigate the increased and diverse health requirements presented.

Previous research on expatriate adjustment has largely overlooked the healthcare sector, focusing instead on other industries [6,7]. This gap highlights the need for a comprehensive study on the acculturation of healthcare expatriates in Qatar, considering age, gender, Arabic proficiency, duration of residence, occupational roles, and social support networks [7,8].

This study provides a comprehensive exploration of the factors influencing expatriate adjustment in Qatar’s healthcare sector, with a focus on cultural differences, workplace dynamics, and social support. Specifically, it examines key variables such as age, gender, nationality, language skills, and social assistance. The insights gained from this research are vital for developing strategies that facilitate smoother transitions for expatriate healthcare workers, enhancing the operational efficiency of Qatar’s healthcare system in the post-World Cup context. By measuring adjustment using both qualitative and quantitative methods, this study aims to offer a detailed understanding of the determinants shaping expatriates' adjustment experiences. The findings will contribute valuable insights for policy formulation and program development, promoting successful integration into both social and professional environments, thereby improving the overall effectiveness and performance of the healthcare workforce.

## Materials and methods

Study design

This analytical cross-sectional study aimed to evaluate factors influencing expatriate adjustment among healthcare professionals in Qatar. Conducted as part of a master’s thesis, the study received ethical approval from the University of Edinburgh’s Institutional Review Board (IRB), under reference B210352. A structured questionnaire was employed to assess socio-cultural and professional adjustment experiences.

Inclusion and exclusion criteria

The study included expatriate healthcare professionals either currently employed or had previously worked in Qatar (public or private sector) and had a minimum of one year of residency in Qatar, ensuring adequate exposure to the host country’s environment. Exclusion criteria included non-healthcare professionals, nationals of Qatar, and expatriates with less than one year of work experience in Qatar.

Data collection

Data were gathered through a structured survey-based questionnaire, developed after an extensive literature review of expatriate adjustment factors and established adjustment models. The questionnaire was pilot-tested for clarity and relevance. It captured demographic data (e.g., age, gender, nationality), professional details (e.g., job role, tenure in Qatar), socio-cultural factors (e.g., Arabic language proficiency, social support networks), and adjustment and social support scales using a five-point Likert scale.

The survey was hosted on SurveyMonkey, a secure online platform, and participants were recruited via purposive sampling through expatriate-focused WhatsApp groups. Group administrators shared the survey link, and weekly reminders were sent during the data collection period, which lasted from October 1 to December 31, 2022. Participation was voluntary, anonymous, and uncompensated. For data cleaning, incomplete or inconsistent responses were excluded through listwise deletion.

Statistical analysis

Data were analyzed using IBM SPSS Statistics 20 (IBM Corp., Armonk, NY, USA). Descriptive statistics summarized demographic characteristics. Relationships between demographic variables, job-related characteristics, Arabic language proficiency, social support, and expatriate adjustment were examined using cross-tabulations and Chi-square tests. A p-value of <0.05 was considered statistically significant. Sensitivity analysis focused on key variables such as Arabic language proficiency and duration of residency to evaluate their influence on adjustment outcomes.

## Results

A total of 199 healthcare professionals participated in the survey, yielding a response rate of 199 (51.69%). Among the 199 healthcare professionals surveyed, 91 (45.7%) were male, while 108 (54.3%) were female. These gender proportions indicate a relatively balanced representation of both sexes within the expatriate healthcare workforce in Qatar. The majority of the participants were in the 35-44 age group, with 77 (38.7%) participants, followed by the 45-54 age group, which constituted 68 (34.2%) of the sample, while those aged 24-34 accounted for 40 (20.1%). The top nationalities represented were Filipino 46 (23.1%), Indian 34 (17.1%), and Egyptian 11 (5.5%). This diversity highlights the multicultural nature of the healthcare sector in Qatar, with professionals hailing from diverse backgrounds and geographic locations.

The demographic characteristics of the participants are detailed in Table 1. The length of stay in Qatar varied among participants, with the highest proportion (78 (39.2%)) having resided in Qatar for four to seven years, followed by those who had stayed for one to three years (71 (35.7%)). Those who have been in Qatar for eight to 10 years and more than 10 years constituted 20 (10.1%) and 27 (13.6%), respectively. A smaller percentage (three (1.5%)) had lived in Qatar for less than a year. This distribution highlights the diverse range of experience durations among expatriate healthcare professionals. The majority of respondents held professional roles, primarily as nurses and doctors, comprising 83.9% (167) of the sample. A smaller proportion held managerial positions (19 (9.5%)), while those in clerical roles constituted 6.5% (13) of the sample. This distribution reflects the predominance of clinical professionals in the expatriate healthcare workforce. Arabic language proficiency among the expats was reasonable. The majority of the participants had basic language skills (116 (58.3%)), while 73 (36.7%) were fluent in Arabic. A smaller percentage had conversational skills (seven (3.5%)), and only three (1.5%) reported having no Arabic language proficiency.

**Table 1:** Socio-demographic characteristics of expatriate healthcare workers in Qatar The table summarizes socio-demographic characteristics of expatriate healthcare workers in Qatar, including gender, nationality, age, duration of stay, job role, and Arabic language proficiency. It presents counts, percentages, chi-square values, and p-values to assess associations between characteristics and adjustment levels. Count: Number of individuals per category, Percentage (%): Proportion within the total sample. Chi-Square: Test statistic for associations, P-value: Significance of associations (<0.05 indicates significance).

Demographic characteristics	Count	Percentage (%)	Chi-square value	P-value
Gender			2.155	0.032
Male	91	45.7		
Female	108	54.3		
Nationality			15.487	0.047
Egyptian	11	5.5		
India	34	17.1		
Jordan	14	7		
Pakistan	21	10.6		
Palestine	7	3.5		
Philippines	46	23.1		
Sudan	12	6		
Syria	8	4		
UK	34	17.1		
USA	12	6		
Age			16.437	0.005
24-34	40	20.1		
35-44	77	38.7		
45-54	68	34.2		
55-64	11	5.5		
65 and over	3	1.5		
Duration of stay			11.357	0.021
Less than 1 year	3	1.5		
1-3 years	71	35.7		
4-7 years	78	39.2		
8-10 years	20	10.1		
More than 10 years	27	13.6		
Job Role			5.732	0.056
Professional	167	83.9		
Manager	19	9.5		
Clerical	13	6.5		
Arabic language proficiency			8.125	0.045
None	3	1.5		
Basic	116	58.3		
Conversational	7	3.5		
Fluent	73	36.7		

The factors influencing the adjustment of expatriate healthcare workers in Qatar are outlined and summarized in Table 2. The analysis suggests that gender is not associated with adjustment levels (p = 0.21). Both male and female healthcare workers exhibit diverse adjustment experiences, with no pronounced gender-related differences. Similarly, the expatriate’s nationality was not associated with adjustment level, the differences do not reach statistical significance (p = 0.167). Some nationalities, such as Jordanians, display a higher percentage of individuals who are Extremely Adjusted (50.00%). However, nationality alone does not account for variations in adjustment experiences.

**Table 2:** Associations between adjustment levels and demographic variables among expatriate healthcare workers in Qatar This table presents the associations between adjustment levels and various demographic characteristics of expatriate healthcare workers in Qatar. The data are shown as frequencies (N) and percentages (%). The adjustment levels are categorized as Extremely Disadjusted, Disadjusted, Neither Adjusted nor Disadjusted, Adjusted, and Extremely Adjusted. The p-values presented in the table were calculated using the Chi-square test to determine the statistical significance of associations between categorical variables and levels of adjustment. The Chi-square statistic (χ²) is displayed alongside the p-value in a separate column to provide additional context for interpreting the results. A p-value less than 0.05 was considered statistically significant, indicating a meaningful association.

Category	Extremely Disadjusted	Disadjusted	Neither Adjusted nor Disadjusted	Adjusted	Extremely Adjusted	Chi-square value	P-value
Gender						1.57	0.21
Male	6 (6.6%)	10 (11%)	32 (35.2%)	26 (28.6%)	17 (18.7%)		
Female	5 (4.6%)	8 (7.4%)	26 (24.1%)	38 (35.2%)	31 (28.7%)		
Nationality						13.31	0.167
Egyptian	1 (9.1%)	1 (9.1%)	4 (36.4%)	4 (36.4%)	1 (9.1%)		
Indian	1 (2.9%)	4 (11.8%)	10 (29.4%)	12 (35.3%)	7 (20.6%)		
Jordanian	1 (7.1%)	1 (7.1%)	2 (14.3%)	3 (21.4%)	7 (50%)		
Pakistani	0 (0%)	0 (0%)	2 (9.5%)	12 (57.1%)	7 (33.3%)		
Age						18.47	0.001
24–34	2 (5%)	4 (10%)	12 (30%)	18 (45%)	4 (10%)		
35–44	2 (2.6%)	6 (7.8%)	17 (22.1%)	28 (36.4%)	24 (31.2%)		
45–54	5 (7.4%)	6 (8.8%)	25 (36.8%)	15 (22.1%)	17 (25%)		
55–64	0 (0%)	2 (18.2%)	4 (36.4%)	2 (18.2%)	3 (27.3%)		
65 and over	2 (66.7%)	0 (0%)	0 (0%)	1 (33.3%)	0 (0%)		
Lived in Qatar						8.77	0.11
Less than 1 year	1 (33.3%)	0 (0%)	1 (33.3%)	1 (33.3%)	0 (0%)		
1–3 years	3 (4.2%)	3 (4.2%)	16 (22.5%)	30 (42.3%)	19 (26.8%)		
4–7 years	3 (3.8%)	10 (12.8%)	21 (26.9%)	25 (32.1%)	19 (24.4%)		
8–10 years	3 (15%)	2 (10%)	9 (45%)	2 (10%)	4 (20%)		
More than 10 years	1 (3.7%)	3 (11.1%)	11 (40.7%)	6 (22.2%)	6 (22.2%)		
Job Title						4.68	0.098
Professional (nurses/doctors)	9 (5.4%)	13 (7.8%)	46 (27.5%)	57 (34.1%)	42 (25.1%)		
Manager	2 (10.5%)	4 (21.1%)	4 (21.1%)	6 (31.6%)	3 (15.8%)		
Clerical role	0 (0%)	1 (7.7%)	8 (61.5%)	1 (7.7%)	3 (23.1%)		
Arabic language proficiency						7.81	0.053
None	0 (0%)	0 (0%)	3 (100%)	0 (0%)	0 (0%)		
Basic	7 (6%)	9 (7.8%)	26 (22.4%)	47 (40.5%)	27 (23.3%)		
Fluent	3 (4.1%)	8 (11%)	25 (34.2%)	17 (23.3%)	20 (27.4%)		

Age appears to have a significant association with the adjustment levels (p = 0.001). Younger healthcare professionals, particularly those aged 24-34 and 35-44, exhibit more balanced distributions across adjustment categories. In contrast, the 65 and over age group primarily consists of Extremely Disadjusted individuals, indicating that younger expatriates tend to adapt better to their new environment. Similarly, the duration of stay in Qatar demonstrates that those who have lived in Qatar for “four to seven years” and “one to three years” appear to be more Adjusted or Extremely Adjusted compared to other groups, although this trend is not statistically significant (p = 0.11). However, healthcare workers with eight to 10 years of experience report a higher proportion of Extremely Adjusted individuals, while those with less than one year exhibit a higher proportion of Extremely Disadjusted individuals.

Job profile does not exhibit a statistically significant association with adjustment levels (p = 0.098). Nevertheless, healthcare professionals in professional roles (Nurses and Doctors) tend to adjust better than those in managerial and clerical roles, as suggested by the higher percentages in the Adjusted and Extremely Adjusted categories.

**Figure 1:**
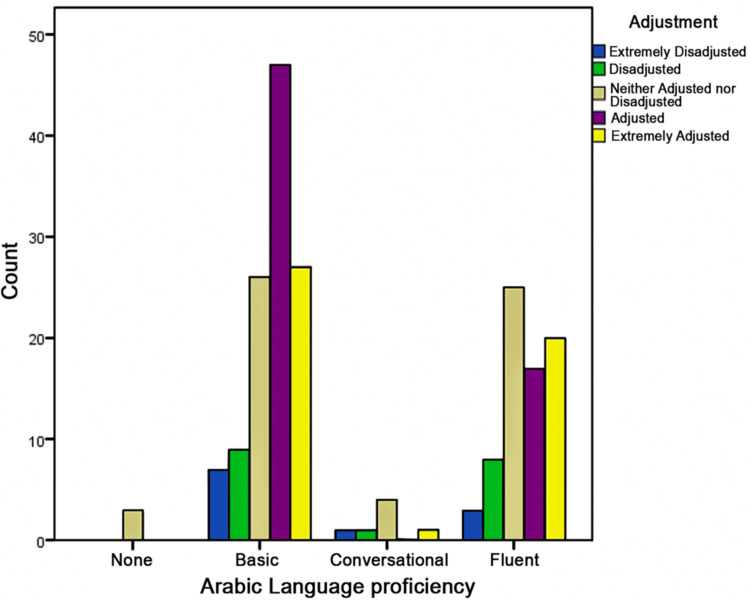
Adjustment level by Arabic language proficiency This bar chart shows adjustment levels among expatriate healthcare professionals by Arabic proficiency: None, Basic, Conversational, and Fluent. The y-axis shows participant counts, with adjustment levels color-coded as: Blue: Extremely Disadjusted, Green: Disadjusted, Gray: Neither Adjusted nor Disadjusted, Purple: Adjusted, Yellow: Extremely Adjusted Higher Arabic proficiency (Conversational and Fluent) correlates with better adjustment levels

Arabic language proficiency (Figure 1) plays a notable role in adjustment (p = 0.053). Individuals with “Fluent” proficiency exhibit better adjustment, with a higher proportion falling into the Adjusted and Extremely Adjusted categories. Furthermore, it was observed that social support strongly influences adjustment levels (p = 0.000). Healthcare workers with “No one support” are more likely to be Extremely Disadjusted, whereas those with “Many would support” or “several would support” primarily belong to the Extremely Adjusted category.

**Figure 2:**
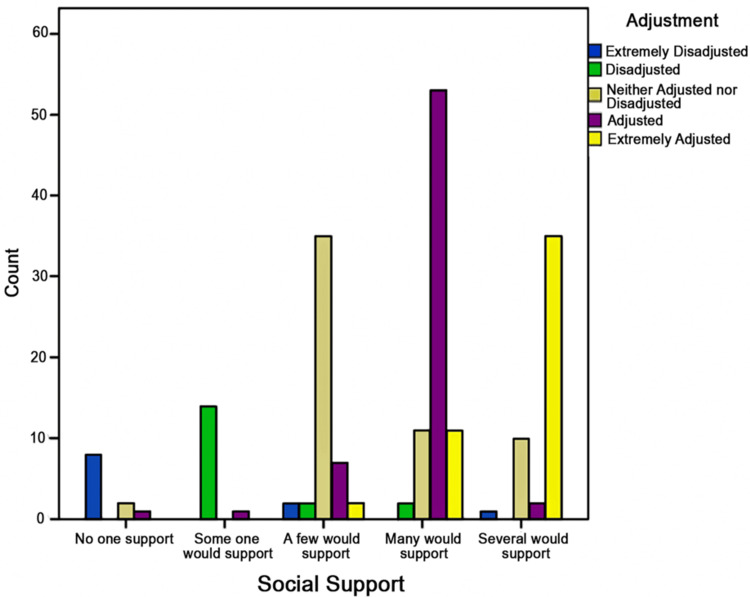
Adjustment level by social support This bar chart illustrates adjustment levels among expatriate healthcare professionals based on social support categories: No one support, Someone would support, A few would support, Many would support, and Several would support. The y-axis represents participant counts, with adjustment levels color-coded as: Blue: Extremely Disadjusted, Green: Disadjusted, Gray: Neither Adjusted nor Disadjusted, Purple: Adjusted, Yellow: Extremely Adjusted Higher levels of social support (Many and Several would support) are associated with improved adjustment outcomes.

Social support was observed to have a profound impact (Figure 2) on the adjustment levels of individuals, confirming the statistical significance of this relationship (p < 0.001). The data reveals a stark contrast in adjustment outcomes: individuals without support predominantly experience extremely disadjustment, while those with substantial social support-categorized as having “Several would support”-tend to be extremely adjusted. This trend underscores the critical role of social networks in facilitating a successful transition and integration into a new professional and cultural milieu.

## Discussion

The study provides a useful understanding of the healthcare workers' adjustment experiences in Qatar and connects with the existing literature on cultural and professional adjustment in healthcare settings. The findings suggest that age and social support significantly influence the adjustment of expatriate healthcare workers in Qatar. Younger individuals and those with strong social networks show better adjustment. While gender, job title, and nationality do not significantly impact adjustment. Language proficiency, particularly in Arabic, also plays a critical role, highlighting the importance of cultural and linguistic integration in the adjustment process.

This study provides a comprehensive analysis of the factors influencing adjustment levels among healthcare workers in Qatar, corroborating findings from similar research [2,3]. The finding highlights the complex relationship of age, duration of stay, language proficiency, and social networks in adjustment to a multicultural and multilingual milieu, highlighting the importance of comprehensive support mechanisms [7,8]. Such insights are pivotal in formulating policies and practices designed to improve the social integration and occupational experience of expatriate healthcare professionals in Qatar, thereby enhancing the overall efficacy and quality of rendered healthcare services.

Gender and job title, while examined, do not exhibit a notable effect on adjustment levels, suggesting that these variables do not independently predict the ease with which expatriates acclimate to the Qatari healthcare sector. This finding may reflect a broader cultural shift towards gender equity and a flattening of hierarchical structures within international healthcare environments. Other studies have observed that female healthcare workers reported superior adjustment levels compared to their male counterparts is particularly salient [9]. This disparity may stem from gender-specific differences in coping mechanisms and the availability and utilization of social support systems. Females, potentially possessing more robust social networks may adopt distinct strategies to acclimate to new environments, resulting in more effective adjustment [10]. Nevertheless, this gender-based variation merits further scholarly scrutiny to unravel the contributory factors, whether personal or professional.

Regarding the influence of age on adjustment, the findings indicate that this variable, although significant, does not demonstrate a linear relationship with adjustment levels. This suggests that adjustment to a novel professional setting in Qatar is influenced by an array of elements, with age being just one of the components [2,11]. The disparate challenges and benefits faced by younger and older workers necessitate a deeper understanding [12]. These findings have profound implications for policymaking and support program development for expatriate healthcare professionals in Qatar. They emphasize the importance of age-sensitive integration strategies and the imperative of constructing robust support networks that foster interpersonal connections and community building [13].

The nationality of the participants is an additional factor impacting their adjustment in Qatar’s healthcare sector. Although the correlation is not significant statistically, certain national groups appear to adjust with varying degrees of success. Expatriates from Jordan and the USA are predominantly in the higher adjustment levels, which may be attributable to socio-cultural similarities or differences, international relations, or pre-existing community support within Qatar. Conversely, Sudanese expatriates display a trend toward lower adjustment levels, which is surprising as they exhibit similarities in cultural or linguistic qualities. There may be other factors such as socio-economic influences not captured by the data. The lack of a strong correlation between nationality and adjustment echoes the findings from other multicultural environments, suggesting a uniformly inclusive experience across different nationalities in Qatar's healthcare sector [12,14]. Qatar's healthcare environment, with its multicultural framework, appears to offer a consistent experience across nationalities, potentially reflecting successful organizational strategies in fostering an inclusive work atmosphere and mitigating the influence of nationality on adjustment [14]. Additionally, local security and stability emerge as critical factors shaping migration decisions, varying by respondents' regional backgrounds. These findings underscore the significance of economic factors in migration choices and highlight the effectiveness of survey experiments in exploring complex migration behaviors [15].

Arabic proficiency significantly influences adjustment (p = 0.053), placing non-Arabic speakers in a unique intermediate adjustment category. This suggests language barriers might encourage proactive cultural engagement, leading to a more complex form of adjustment. Other studies have noted a marginally significant relationship between Arabic language proficiency and adjustment level [16]. This indicates that while language skills are pertinent, they are not the sole determinants of successful adjustment. The prevalence of English as a lingua franca in the healthcare sector and the adaptability of healthcare workers in surmounting language barriers could explain this observation [17-19].

The length of stay in a foreign country can significantly aid in the adjustment process for expats. The adjustment involves several dimensions including cognitive, affective, and behavioral aspects, and it spans across different domains such as work, culture, and personal life. The expatriate experience typically unfolds in four phases [20]. The Preparation phase involves getting ready for the move, often filled with anticipation and planning. In the Honeymoon phase, upon arrival, expatriates usually experience excitement and curiosity about their new surroundings. This is followed by the Culture Shock phase, where the initial enthusiasm may give way to frustration and anxiety due to cultural differences and challenges like language barriers. Finally, in the Adjustment phase, expatriates gradually become more comfortable and adept at navigating the new culture, leading to a more settled and satisfying experience [20]. Immersing oneself in the local culture and breaking out of one's comfort zone are crucial steps in this process.

Social support significantly influenced adjustment outcomes (p < 0.001). Lack of support (“No one support”) strongly correlated with “Extremely Disadjusted” outcomes (72.7%). In contrast, having substantial support (“Many would support” or “Several would support”) was associated with being “Extremely Adjusted” (68.8% and 72.9%, respectively). This highlights the crucial role of social networks in successful adjustment, indicating that individuals without support often face significant misadjustment, while those with robust support systems tend to adjust exceptionally well. The data emphasize the critical importance of social support in effective integration into new professional and cultural settings [21].

In summary, this research offers valuable insights into the factors that influence the adjustment of expatriate healthcare workers in Qatar. The findings highlight the importance of considering variables such as age, social support, gender, nationality, and language proficiency in healthcare policy and management. By addressing these factors, healthcare administrators in Qatar can improve the integration and performance of their expatriate workforce, contributing to the overall quality and effectiveness of healthcare services in the country’s diverse and evolving healthcare environment.

Limitations and recommendations

This study has several limitations that should be considered. While the sample was diverse, it may not fully represent the entire healthcare workforce in Qatar, as participants were primarily recruited through a limited number of expatriate-focused WhatsApp groups. This recruitment strategy could introduce selection bias, potentially resulting in the underrepresentation or overrepresentation of certain demographics. Additionally, the reliance on self-reported data raises concerns about response bias, which could affect the validity of the findings.

Moreover, the study’s cross-sectional design provides only a snapshot of expatriate adjustment, potentially overlooking the evolving nature of cultural adaptation over time. A longitudinal study would offer a more detailed understanding of the dynamic processes involved in adjustment, allowing for a clearer view of how adjustment levels shift and what drives these changes. Additionally, while the study identifies key patterns, a more sophisticated analysis is needed to explain non-significant trends and identify the factors contributing to them. We also acknowledge the limitations in causal inference due to the cross-sectional design, which restricts our ability to conclude cause-and-effect relationships.

The analysis of key factors such as language proficiency and psychological variables, including stress and culture shock, was limited. A more in-depth exploration of these elements is recommended to better understand their role in the expatriate experience. Incorporating qualitative methodologies, such as interviews or focus groups, could provide richer insights into the personal and emotional aspects of expatriate adjustment.

Furthermore, the study’s scope was somewhat narrow, as it did not fully consider the potential impact of organizational policies and workplace culture on adjustment. The sampling strategy, while convenient, may also have introduced biases that affected the interpretation of results. Future research should adopt a more comprehensive approach, examining the interplay between individual, organizational, and cultural factors to gain a holistic view of expatriate adjustment in healthcare settings. This would ensure a more balanced interpretation, particularly in areas where the data does not show statistical significance.

## Conclusions

This study reveals several factors of adjustment among expatriate healthcare workers in Qatar. Notably, it underscores that gender plays a significant role, with female healthcare workers generally reporting better adjustment. However, the influences of age and job titles on adjustment are found to be complex and nonlinear. Contrary to common perceptions, nationality and duration of stay in Qatar are shown to have less influence on the adjustment process. Instead, the study emphasizes the critical importance of social support networks in facilitating expatriate healthcare workers' successful adjustment to the multicultural environment of Qatar. These findings have important implications for policymakers and healthcare administrators, highlighting the need for tailored support systems that can enhance the well-being and professional integration of expatriate healthcare workers, ultimately contributing to the overall quality of healthcare services in such diverse settings.

## Appendices

**Table 3:** Questionnaire on the adjustment of expatriates The questionnaire was developed after an extensive literature review using established expatriate adjustment models [6,7]. Proper attribution is included in Materials & Methods, and no permissions were required as the content is based on publicly available models.

Category	Question	Response options
Demographics and language proficiency		
Gender	[Dropdown: Male, Female, Prefer not to say]	
Age (years)	[Dropdown: 24-34, 35-44, 45-54, 55-64, 65 and over]	
Nationality	[Dropdown: Egypt, India, Jordan, Pakistan, Palestine, Philippines, Sudan, Syria, UK, USA]	
Occupational level	[Dropdown: Senior Executive, Middle Management, Junior Staff, Clerical]	
Arabic language ability	[Dropdown: None, Basic, Conversational, Fluent]	
Experience in the company	Rate on a five-point scale from “No one” to “Many” for the following:	
Do you have a companion for necessary activities?		
Is there someone to check on your well-being?		
Do you have a source for orientation information?		
Is there assistance with local regulations?		
Can someone clarify complex situations for you?		
Does someone inform you of local prohibitions?		
Is there guidance for unfamiliar tasks?		
Do you receive help in understanding ambiguities?		
Is there support for communication challenges?		
Does someone help you grasp the local culture and language?		
Are you informed about your options?		
Do you have company for outings without obligation?		
Adjustment in Qatar	Rate on a five-point scale from “Extremely Adjusted” to “Extremely Unadjusted” for the following:	
Overall living conditions		
Quality of housing		
Access to food and shopping		
Affordability of living		
Availability of entertainment and leisure		
Quality of healthcare services		
Clarity of job duties		
Clear performance metrics		
Management roles clarity		
Social life with locals		
Daily interactions with locals		
Socializing with locals outside work		
Ease of communication with locals		
Open-ended Questions	Describe challenges faced as an expat.	
	Suggestions for improvements for new expats.	
